# P045: A multimodal strategy to improve the application of decolonisation treatment for the resident carrier of MRSA in nursing homes in Switzerland

**DOI:** 10.1186/2047-2994-2-S1-P45

**Published:** 2013-06-20

**Authors:** L Qalla-Widmer, C Bellini, G Zanetti, C Petignat

**Affiliations:** 1 Unité HPCI, CHUV, Lausanne, Switzerland

**Keywords:** MRSA, prevalence, implementation, protocol, decolonisation treatment, resident, nursing home

## Introduction

In order to evaluate the methicillin resistant *Staphyloccus aureus* (MRSA) carriage rate, the cantonal unit for Hygiene, Control and Prevention of Infections (HPCI unit) undertook, in 2010, a survey of prevalence in104/157 nursing homes of canton Vaud (Western Switzerland). In the contest of survey, a project on the implementation of decolonisation protocol was conducted.

## Objectives

The objective of this strategy was to improve the success rate of decolonisation by insuring good practice in the application of treatment of the MRSA carrier resident by the health care workers in nursing homes.

## Methods

To foster adhesion to the protocol by the nursing staff, a strategy based on the creation of teaching aids (flyers, video) was to associated to the written protocol. A training course on Standard Precautions based on the management of the MRSA carrying resident for the nursing staff was provided. Audits of practices to observe the quality of the application of treatment by nursing staff were conducted. The MRSA carriers underwent a 5-day topical decolonisation (nasal mupirocine and chlorhexidine disinfection of skin and pharynx) combined with environnemental disinfection. The treatment was repeated once in case of failure. The survey took place between June and December 2010. Residents with either urinary or wound (stage IV) colonisation were excluded.

## Results

Of the 2941 screened residents, 356 were MRSA carriers, the mean prevalence was 8.9%. Because of urinary, wound colonisation or death, 92 residents were excluded. Successfull decolonisation was observed in 264 residents (60%) this is higher rate than in acute care facilities. Adherence to protocol by nursing staff was observed (accordind to establisched criteria) during audits and the observance rate was 94%.

## Conclusion

Multimodal strategy based on coaching nursing staff and teaching aid availability is an effective way to optimize the adherence to the decolonisation protocol and to improve success rate of decolonisation.

## Disclosure of interest

None declared

